# Emerging technologies’ role in reducing under-five mortality in a low-resource setting: Challenges and perceived opportunities by public health workers in Makonde District, Zimbabwe

**DOI:** 10.1177/13674935231189790

**Published:** 2023-07-18

**Authors:** John Batani, Manoj Sewak Maharaj

**Affiliations:** 1 School of Management, IT and Governance, College of Law and Management, 56394 University of KwaZulu-Natal, Durban, South Africa; 2 Faculty of Engineering and Technology, Botho University, Maseru, Lesotho

**Keywords:** child mortality, infant mortality, nurse–child interaction, care, information technology, SDG 3

## Abstract

Under-five mortality (U5M) remains a global challenge, with Sub-Saharan Africa being the hardest hit. The coronavirus disease 2019 (COVID-19) has strained healthcare systems, threatening to reverse current gains in U5M health outcomes. It threatened progress made towards achieving United Nations Sustainable Development Goal 3 due to its strain on healthcare systems, resource reassignment and its prioritisation by health authorities globally. Low-resource settings inherently face unique challenges in fighting U5M and providing quality healthcare to under-fives, like understaffing, drug shortages, underfunding, skills gaps and lack of specialised healthcare equipment, contributing to high U5M rates. This study explored public health facilities’ challenges in reducing U5M in a low-resource setting in Zimbabwe and public health workers’ perceptions of emerging technologies’ role in addressing those challenges. Twenty public health workers participated in interviews and a focus group. They perceived emerging technologies (ETs) as a panacea to the challenges by supporting data-driven healthcare, improving follow-up outcomes through automated reminders of medication and clinic visits, aiding diagnosis, continuous monitoring, health education, drug supply monitoring, critical supplies delivery and skills development. In this paper, emerging technology is any information and communication technology that has not been utilised to its full potential in Zimbabwe’s public health domain. Findings indicate that public health workers in Makonde would welcome ETs to improve under-five health and well-being.

## Introduction

Incorporating under-five mortality (U5M) into United Nations Sustainable Development Goals (UN SDGs) (The United Nations, 2015b) signifies its global importance. U5M rate (U5MR) refers to the number of children who die before turning five per every thousand babies born alive (Haley et al., 2017). Sub-Saharan Africa (SSA) has the worst global U5M statistics (United Nations Inter-Agency Group for Child Mortality Estimation (UN IGME), 2019, 2020). It failed to achieve targets set in the expired Millennium Development Goals (MDGs) that were replaced by United Nations Sustainable Development Goals (UN SDGs) in 2015 (United Nations Economic Commission for Africa, 2015). SSA’s adoption of emerging technologies (ETs) for paediatric care is still low, with mainly short messaging services, mobile apps and websites being used for antenatal and paediatric care (Batani and Maharaj, 2022a).

Zimbabwe is amongst countries with high U5MR (UN IGME, 2021), despite some strides made through interventions like prevention of mother-to-child transmission of human immunodeficiency virus and free public healthcare for under-fives (Zimbabwe National Statistics Agency (ZIMSTAT, 2019)). Despite the free healthcare policy, required medicines are sometimes unavailable in remote health facilities, which are also understaffed as health workers shun those areas due to poor working environments and infrastructure (Haley et al., 2017), such as bad roads and network connectivity (Batani et al., 2019; Batani and Maharaj, 2022b).

The coronavirus disease 2019 (COVID-19) pandemic seriously threatens the UN SDG 3 target of reducing U5MR to 25 per 1000 live births (The United Nations, 2015a). COVID-19 has exacerbated the problems due to health workers’ reassignment to COVID-19 programmes, travel restrictions, prioritisation of containing the pandemic and missed critical vaccinations by some under-fives (Organisation for Economic Co-operation and Development (OECD), 2020; The Royal College of Paediatrics and Child Health (RCPCH), 2020). Emerging technologies provide hope through unprecedented opportunities and healthcare benefits and improvements, like remote consultations, provision of trusted health information (Mbunge et al., 2022), health education (Sharma and Gupta, 2017), remote patient monitoring, pervasive healthcare (Mbunge, Muchemwa, et al., 2021), disease trends prediction and monitoring and disease prevention, as witnessed in their deployment against COVID-19 (Mbunge, Dzinamarira, et al., 2021). Health literacy positively impacts health outcomes (Otten et al., 2023); hence, health education is important.

In the last decade, digital health studies in Zimbabwe have mainly focused on general health and not on reducing U5M (Batani and Maharaj, 2022b). Examples include factors influencing electronic health adoption by medical doctors (Furusa and Coleman, 2018), assessing Internet of medical things adoption readiness (Chipambwa and Terera, 2016) and digital health’s potential to enhance equity and quality of care (Bishi et al., 2017). The relationship between mother’s education and child mortality in Zimbabwe (Grépin and Bharadwaj, 2015) and the association between mothers’ health-seeking behaviour and U5M in Zimbabwe (Chadoka-Mutanda and Odimegwu, 2017) have also been studied. In this study, an emerging technology is any information and communication technology that has not been utilised to its full potential in Zimbabwe’s public health domain.

Zimbabwe’s U5MR is nearly twice (54 per 1000 live births) the UN SDG 3 target (UN IGME, 2019; 2020); however, research into public health workers’ perceptions in resource-poor communities towards addressing the attendant challenges is not evident. While ETs provide some hope for attaining the UN SDGs (Sachs et al., 2016), policymakers must understand public health workers’ perceptions of implementing such technologies in healthcare, especially in paediatrics.

There is a dearth of literature on public healthcare professionals’ perceptions of ETs’ role in reducing U5M in resource-constrained settings. This study explored emerging challenges in reducing U5M in a resource-constrained setting and health workers’ perceptions on the role of ETs in addressing those challenges, hence, helping reduce U5MRs.

### Aim

The aim of this study was to explore challenges faced in fighting U5M and public health professionals’ perceptions on the potential role of emerging technologies in addressing challenges in Zimbabwe’s public health.

## Materials and methods

The study employed an exploratory research design using semi-structured interviews and a focus group for data collection. Researchers electronically collected data due to COVID-19-induced restrictions.

### Inclusion and exclusion criteria

The study targeted public health professionals from Makonde District, Zimbabwe. Qualifying participants were health professionals who were paediatricians, midwives or worked with under-fives at a public health institution in Makonde District. Researchers included the Provincial Paediatric Specialist, Provincial Medical Director and District Medical Officer to gain management’s insights. Researchers excluded public health professionals who did not work in paediatric wards or with children under five. However, this exclusion criterion only applied to Chinhoyi Provincial Hospital, where health workers specialise. Health workers at satellite clinics did not specialise; thus, they all worked with under-fives and qualified for inclusion.

### Participants selection

Purposive and snowball sampling techniques were used. Potential participants were approached through WhatsApp or electronic mail. Sample adequacy was based on saturation (Vasileiou et al., 2018). Four nurses and one doctor refused to participate, citing that they were busy with COVID-19 vaccination programmes. There were no dropouts.

### Setting

We conducted the study in Makonde District’s public health facilities in Zimbabwe and drew participants from nine clinics and one hospital. Interviews were conducted during respondents’ free time. Participation was from home or work but away from non-participants. Data were collected between January and March 2022.

### Data collection

Semi-structured interviews and a four-participant focus group were administered using Google Meet, Microsoft Teams, Zoom and WhatsApp calls, depending on respondents’ preferences. Discussions were audio and video recorded with participants’ consent. Respondents were given a copy of the interview questions in advance. No repeat interviews were conducted.

### Ethical considerations

University of KwaZulu-Natal’s Human and Social Sciences Research Ethics Committee (Approval number: HSSREC/00002132/2020) and the Medical Research Council of Zimbabwe (Protocol Number: MRCZ/A/2782) ethically cleared the study. Permission letters were obtained from Mashonaland West’s Provincial Medical Director (PMD) and Makonde Rural District Council (RDC)’s Chief Executive Officer since all clinics in Makonde Rural are under the Makonde RDC. Before participation, participants were sent a consent form, and the researcher explained the research details before they returned a signed copy of the form. They could withdraw at any time with no consequences to them.

### Data analysis

Recordings of interviews and a focus group were translated verbatim using QSR International’s NVivo 12 software transcription functionality and confirmed by listening to the recordings and verifying if the transcript was correct. Data were coded and themes derived. Researchers used pseudonyms to anonymise respondents’ names before analysis. Initial codes were identified through line-by-line coding. Themes were coded as nodes, bundling responses related to each of them. Each transcript was individually coded before merging and refinement. Nodes were recursively refined, merging similar ones. Coding started after performing 12 interviews, and further interviews were conducted to clarify concepts until saturation was reached.

## Results

Twenty public healthcare professionals from Makonde District participated in online interviews and a focus group, and their distribution by health facility is shown in Table 1. An interview lasted an average of 30 minutes. There were 25 per cent more female participants than males. Of the twenty respondents, two were health information officers, as shown in Table 2. Several themes emerged from data analysis, as presented below. Under each theme, the authors describe the challenge and emerging technologies’ perceived role in reducing U5MR.

**Table 1.** Distribution of healthcare respondents by health facility and gender.

Health facility	Gender	Count	Total and % by health facility
Chinhoyi Provincial Hospital (includes 1 respondent from Sub-national level)	Female	5*	8 (40%)
	Male	3	
Kenzamba Clinic	Female	1	1 (5%)
Mutala Clinic	Male	1	1 (5%)
Matoranjera Clinic	Female	2	2 (10%)
Portlet Clinic	Female	2	2 (10%)
Godzi Clinic	Male	1	1 (5%)
Doma Clinic	Male	1	1 (5%)
Umboe Clinic	Female	1	1 (5%)
Runene Clinic	Female	1	1 (5%)
Kosana Clinic	Male	1	1 (5%)
Total	Female	**13**	**13 (65%)**
	Male	**7**	**7 (35%)**

**Table 2.** Distribution of participants by gender and role.

Designation	Gender				Count and % of N
	Male		Female		
	Count	% of N	Count	% of N	
Provincial Specialist Paediatrician	1	5.0%	0	0.0%	1 (5%)
Midwife	1	5.0%	4	20.0%	5 (25%)
Registered General Nurse	1	5.0%	4	20.0%	5 (25%)
Paediatrician	0	0.0%	1	5.0%	1 (5%)
District Medical Officer	1	5.0%	0	0.0%	1 (5%)
Health Information Officer	1	5.0%	0	0.0%	1 (5%)
District Health Information Officer	0	0.0%	1	5.0%	1 (5%)
Primary Care Nurse	1	5.0%	1	5.0%	2 (10%)
Doctor	1	5.0%	2	10.0%	3 (15%)
Total	**7**	**35%**	**13**	**65%**	**100%**

### Access to health services

Many children under five in Makonde District face challenges accessing health services. These include transport problems due to bad roads, limited health facilities and long distances to health facilities, leading to delayed access to healthcare. Delayed access to healthcare may lead to deterioration and worsening of the babies’ conditions and even developing complications. Moreover, the long distances to health facilities have led to increased home births, which can delay identifying neonatal illnesses and conditions requiring urgent medical attention. Delayed access to healthcare can have detrimental consequences, including death.

The challenges we face in reducing under-five mortality include limited health facilities and distances travelled by people from their homesteads to health facilities which cause more delays, leading to death. There is a lack of transport. Some people use scotch carts. Some do not even have cattle or scotch carts, contributing to infants’ deaths. There are also no proper bridges. They may fail to cross rivers to visit health facilities, especially during the rainy season (RGN#2).

Others concurred that delayed access to healthcare contributed to high U5MRs through delayed identification of abnormalities:

Parents take long to bring their babies to us, delaying them from getting medical examination and referral to the next level for assistance. One mother didn’t come with her baby post-delivery at three days, seven days and every other recommended visit. She only came at ten weeks, and the baby had an abnormal head size (RGN#13).

We have some reported cases of home deliveries and late bookings, which lead to complications and late identification of abnormalities (PCN#9).

Poor roads and bad terrains delay critical supplies’ delivery to clinics from the provincial pharmacy. ETs like the Internet of medical things (IoMT), telemedicine, telediagnosis, drones and telehealth were identified as potential solutions to enhancing health access. Drones were cited as helpful in delivering urgently needed drugs, while IoMT, wearable devices and sensors help in remotely monitoring critical medical conditions amongst under-fives, allowing pervasive healthcare.

Makonde District only has one public hospital where doctors and specialists are stationed. Nurses said they would appreciate using telemedicine to get remote assistance diagnosing complicated cases to help under-fives get early assistance and minimise referrals to the hospital. A nurse based at a satellite clinic weighed in on the potential role of ETs in reducing U5MRs in Makonde:

It will reduce very much with a very big margin .… due to these bad terrains that we have, it will be very much helpful if we use telemedicine, connecting with a specialist on a screen so that I will be able to assist children and it will minimise the delays of referral from local clinic to the hospital …. We need to minimise the need for patients to go to town because, most of the time, they won't have money and will just stay at home. We need these technologies to help us have proper diagnosis and disease management and also move with the times (RGN#13).

A primary care nurse at a remote clinic said that drones could help deliver critical medication:

It is just 30 kilometres from here to town, but it takes us over two hours to reach town due to poor roads. The poor roads delay essential drugs’ delivery. Using technologies like drones to deliver essential drugs could allow us to access life-saving medications on time, helping save lives of under-fives (PCN#8).

### Knowledge deficiency

Lack of knowledge leads to failure in seeking timely medical assistance and subsequently trying unproven home remedies. Participants said that some parents lack knowledge of how to take good care of their under-fives, the importance of timely treatment, disease prevention and what to take to health facilities for deliveries, while others use cow dung to dress children’s wounds, causing infection.

Another factor is a lack of knowledge – some women try attending to their babies on their own using methods that are not scientifically proven. For instance, we have had cases of some women applying cow dung to babies’ wounds which even makes the babies susceptible to further infection (RGN#7).

A case of a child who missed recommended check-ups led to complications by the time she sought medical attention 10 weeks after birth. Respondents also noted that lack of knowledge sometimes makes pregnant women fail to get assistance from health facilities.

Some mothers have a knowledge deficit and lack of resources ... For example, a woman gets into labour preterm. They use scotch carts from their homes to a nearby clinic. In that scotch cart, no one knows how to deal with preterm delivery. Sometimes, …, when they get to the clinic, they do not know what to bring [medical consumables] to the clinic for them to be helped …. So when they get to the clinic, they can’t be helped because they do not have the resources. Midwives sometimes ask accompanying relatives to buy razor blades, gloves and so many things, and sometimes they won’t have the money to buy those things. So a woman is attended to late because she did not know what to bring … They did not know what to bring to the hospital, and they did not have transport to come to the hospital ... (RGN#10).

Respondents indicated that ETs could help provide health education on care for under-fives, disease prevention and management methods, and the importance of timely access to healthcare.

Emerging technologies can assist, …, especially with public health education on disease prevention and taking good care of under-fives. Visuals are easier to understand, especially when giving health education. Using technology like TV, social media and electronic billboards to present real videos of preventable diseases of under-fives, parents will have a better understanding compared to only telling them in words. The roles come in simplifying health promotion lessons and visualisation of possible complications that may arise if proper preventive measures are not followed (RGN#3).

### Resource shortages

Respondents lamented resource shortages in Makonde District’s public health facilities. They indicated that sometimes they run out of drugs, and the facilities are under-equipped, especially with respect to diagnosis, resuscitative and other equipment necessary to preserve under-fives’ lives. It was stated that public health facilities get drug deliveries quarterly, the allocation of which is not necessarily based on each clinic’s consumption, leading to early stock-outs in some health facilities. When stock-out happens, health workers ask parents or guardians to go buy medication for their under-fives from the pharmacies in town, but some of the parents will not have money for transport and drugs. Without treatment, some children get worse and even die.

… It will be much easier if we also get it [technology] for drug supply to notify us of drugs that need restocking, unlike having to wait for a routine supply of drugs. Whenever we prescribe drugs, if the drug is about to get finished in stock, it should notify us and notify the responsible people who dispense drugs to clinics. It should reflect on drug usage and clients’ flow. It should inform us on which drugs are more in demand and where? That should help make informed decisions. We have under-fives who will go without some drugs as they will be out of stock (PCN#9).

Healthcare workers perceived ETs as helpful in addressing the challenge of resource shortages through continuous monitoring of drug levels and skills development through remote training. More so, they can get remote assistance with diagnosing complicated cases from specialists at the provincial hospital or anywhere.

Telemedicine and telehealth will be welcome because on the ground right now, you will find out that when we observe some patients, we can see that this is a referral case, but because of financial constraints, they will just go back home. So if such technologies can be introduced, it cuts costs and saves lives as they will focus only on medication, of which we give them drugs for free when we have them (PCN#9).

In cases where drugs are urgently needed to save lives, it was stated that drones could be used to deliver them quickly.

Drones that can be used to deliver medications, for example, in disaster-stricken areas, like what we had in Chimanimani with Cyclone Idai. If we had drones, we could bring medications and food items to those under-fives in need (RGN#10).

### Religious and cultural beliefs

Religious and cultural beliefs are a real cause for concern in enhancing care for under-fives in some areas of Makonde. Some churches regard going to a health facility as a sin and indication of lack of faith in God. Apart from the demonisation of health facilities, many people hold beliefs prohibiting family planning. They then conceive every year, depriving the babies of sufficient breastfeeding times recommended by healthcare professionals.

Again, there is this place around our catchment area where the apostolic sects don’t want to go to the clinic due to religious beliefs. Sometimes we hear reports of under-fives who would have died from measles or chicken pox. And because these people usually have babies every year, the babies won’t have enough breastfeeding times, and the babies will develop kwashiorkor [a severe form of malnutrition]. They don’t come to us for assistance, like plumpy nuts [peanut-based paste in a plastic wrapper to treat severe acute malnutrition] and other health education. The babies won’t have enough breast milk and will develop health problems (RGN#13).

Besides religion, cultural beliefs were also cited as a challenge in the district. It was stated that some women prefer giving birth at home instead of in-facility due to cultural beliefs, as they would want to perform cultural rituals during or just after birth. Moreover, some of them prefer being attended to by traditional or untrained midwives in their villages. Some women are also uncomfortable being helped by male nurses during delivery, prompting them to prefer home deliveries or going to untrained village midwives.

On ETs’ potential role in addressing religious and cultural beliefs, most rural-based nurses noted that they could help debunk some myths and misinformation, but there is a need for technology and law enforcement. They suggested that community radios could help since most people have mobile phones, most of which can connect to radio. However, it was suggested that such radios should present content in the two major local languages – Shona and Ndebele – that are mostly spoken and understood by everyone in the area.

I think the continued use of advertisements and radios, using native languages like Shona and Ndebele, which are mainstream languages in this areas. There is wide radio ownership around here; about 80% own radios. Remember, phones also have radio access. I also estimate that about 70–80% of the people within our catchment area have mobile phones (PCN#9).

Besides education and demystification, respondents perceived ETs, like mobile apps and toll-free numbers, as helpful in anonymous reporting of unimmunised and sick babies whose parents are not taking them to health facilities. This could help health workers, with the help of the police, to visit such homes and administer vaccinations, immunisations and treatments while arresting such parents since it is a crime in Zimbabwe to deny babies access to immunisation.

### Parents’ or guardians’ negligence

It was mentioned that some parents or guardians are negligent and do not take good care of their under-fives following guidelines from health professionals.

Moreover, some parents are negligent; they will say they don’t have money to go to the clinic, yet the services from the local clinics are free. Sometimes they try wrong home remedies, and the cases would have become complicated when they think of coming with the babies. Like malnutrition, I have seen babies who come when their stomachs would have swollen (RGN#13).

Most respondents perceive ETs as useful in anonymously reporting negligent behaviour to the police and nearby health facilities providing health education to promote behaviour change.

We can also give information, for example, to vaccinate children against diseases. This will help prevent disease among children under five because they won’t suffer from them once vaccinated against such diseases. Even if they would suffer from them, it won’t be as bad as if they were not vaccinated. We can even give health information on what to do against certain conditions. For example, we have cases of burns. Children can be burnt, and their parents or guardians start applying anything, like cow dung, on these wounds and by the time the children come for medical help, they will be already infected. So we can give information on basic red cross knowledge on [handling] such conditions so that they will be manageable when the children finally visit the hospital …. (RGN#10).

### Loss to follow-up

Participants revealed that health workers sometimes fail to make follow-ups on under-five patients who would have missed immunisations or defaulted on medication. They said it is difficult to know which child is due for immunisation because they currently use paper records to record that information. However, the respondents believe using technologies would minimise loss to follow-up since they would remind them of under-fives due for immunisation and defaulters on critical medication.

… there is what we call ‘loss to follow-up’; but with databases and emerging technologies, there won’t be any loss to follow-up …. it would be easier for us to pick defaulters ……. So everything is input into a database, making it easier to identify and follow-up on defaulters ….. (DPPS#1)*.*

### Quality of care

Clinics in Makonde are only staffed by nurses as doctors are only stationed at Chinhoyi Provincial Hospital, several kilometres away from most clinics. Nurses indicated that they sometimes face problems diagnosing some ailments in under-fives and then call the District Medical Officer (a doctor) for assistance using their airtime. The respondents believe that ETs, like artificial intelligence (AI), could help them diagnose diseases better and improve the quality of care by minimising misdiagnoses. Participants revealed that at Chinhoyi Provincial Hospital, they are currently using an AI-powdered application for neonates called Neotree to help health practitioners manage illnesses and the growth of babies.

Also, quality of care, for example, AI helps healthcare professionals to make good judgments and manage patients without risking them. That improves the QoC [quality of care] by reducing misdiagnosis and mismanagement of patients because doctors will be consulting this AI for better judgment, reducing human error and the risk of preventable surgeries. Sometimes surgeries are done on patients on whom they were not supposed to be done …. Also, when patients are managed in their homes, for example, in virtual consultation where a patient can ask where they get help, physicians, [and] appointments can be done on their mobile phones, helping manage under-fives as they will come to hospitals early, and there is remote access to services; … people can be told how to treat certain symptoms using these emerging technologies. Such that they can be stabilised and come to hospitals when they are stable and manageable …. (RGN#10)*.*

**Figure 1.**
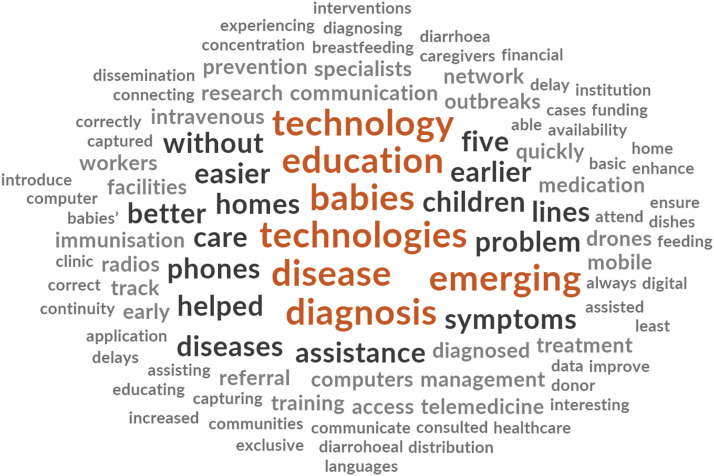
Word cloud for perceived role of emerging technologies.

A word cloud on ETs perceived role in reducing U5MRs is shown in Figure 1.

## Discussion

Reducing under-five mortality is a global challenge, mainly in SSA, where under-fives are 15 times likelier to die than their counterparts in developed countries (UN IGME, 2021). Having failed to achieve MDG targets on U5M, SSA remains on the verge of failing to achieve UN SDG targets on the same outcomes, with COVID-19-induced disruptions worsening the situation (Batani and Maharaj, 2022a). Emerging technologies provide hope for attaining stretch UN SDG targets (Sachs et al., 2016). However, there is a lack of emerging technologies adoption in public paediatric care in SSA, with most digital systems being uni-directional SMS, mobile apps and websites that lack data-drivenness by utilising data collected from routine health information systems (Batani and Maharaj, 2022a). Thus, data-driven paediatric care could enhance U5M outcomes in SSA and other resource-constrained settings (Batani and Maharaj, 2022b). However, mainstream literature is silent on public healthcare professionals’ perceived role of emerging technologies in reducing U5M in resource-constrained countries.

The study identified challenges in reducing U5MR and ETs’ role in addressing them. The challenges include knowledge deficiency, long distances to health facilities, understaffed health facilities, drugs and critical equipment shortages, home births, wrong home remedies and transport problems. ETs’ perceived role in reducing U5MR includes enhancing health education, continuity of care, minimising the need for referrals, urgent supply of critical drugs, remote training, ease of diagnosis and improving patient follow-ups. Emerging technologies’ role in enhancing public health has been discussed by various researchers, albeit not from health professionals’ perspectives and unspecific to reducing U5MRs in resource-constrained environments. Literature supports our findings that emerging technologies strengthen health education and awareness (Alshammari et al., 2017; Sharma and Gupta, 2017), remote, pervasive and personalised care (Mbunge, Muchemwa, et al., 2021), disease diagnosis (Davenport and Kalakota, 2019), remote patients treatment and monitoring (Portnoy et al., 2020) and treatment support (Mbunge, Muchemwa, et al., 2021; Ventola, 2014). However, reducing loss to follow-up is less discussed in existing literature as a role of technology, yet this is crucial in the face of COVID-19-disrupted child immunisation programmes (Batani and Maharaj, 2022a).

Despite positive views on ETs’ role in fighting U5M in the district, public healthcare is not mainly technology-driven as most technologies they wish to have (like telemedicine, AI, electronic billboards, telediagnosis and IoMT) are unavailable. This lack of technology-driven paediatric care in the district’s public health facilities could be explained by underfunding (United Nations Development Program Zimbabwe, 2020), network problems in some areas (Batani et al., 2019), lack of trust and privacy issues (Furusa and Coleman, 2018) and centralisation of digital health policy and decision-making (Batani and Maharaj, 2022b).

Participants appreciated technology’s role in achieving UN SDG 3; however, most had researched these technologies before the interviews, and they never did any course on digital health or digital paediatrics in college. The lack of awareness and exposure to digital paediatrics perhaps justifies the lack of adoption of digital paediatrics in resource-constrained settings like Zimbabwe (Batani and Maharaj, 2023; Furusa and Coleman, 2018). Furthermore, a lack of awareness of ETs’ benefits to paediatric care could cause resistance to such technologies when implemented, as evidenced by healthcare professionals’ resistance to Neotree’s introduction in the district (Batani and Maharaj, 2022b). Due to underfunding and reliance on donors, most digital health interventions in resource-constrained settings, like Neotree (Gannon et al., 2021), end at pilot stages (Batani and Maharaj, 2023). Regardless of positive sentiments on ETs in paediatric care, there could still be impediments to their full adoption, like resistance to change, health policy, funding, computer literacy (Furusa and Coleman, 2018), power outages and unreliable network connectivity.

Implications of these findings are that there are still critical challenges in fighting U5M in the district, which, if not addressed on time, could jeopardise Zimbabwe’s achievement of the UN SDG 3 target of reducing U5MR to 25 per 1000 live births. Yet, these findings also imply that the public health workers in Makonde are ready to accept, adopt and use digital ETs that can help them improve the health and well-being of under-fives, such as telemedicine, drones, virtual reality and artificial intelligence. The national health authorities and donor communities must work on ensuring that Zimbabwe achieves the SDG 3 target on U5M by introducing some of these technologies in public health facilities in Makonde District.

### Study contribution, strengths and limitations

This study enriches the literature by adding public health workers’ views on ETs’ potential role in reducing U5MRs in resource-constrained settings by addressing challenges faced in improving the health and well-being of under-fives. Practically, it provides insights to policymakers and potential partners in the health domain to understand the current challenges and the public health workers’ views on what role technology could play.

This study was limited in not including participants from all the public health facilities in Makonde District. However, participants’ recruitment was stopped after reaching saturation. Saturation is when the responses obtained no longer generate new insights (Bowen, 2008), themes or subthemes (Vasileiou et al., 2018). The researchers decided that saturation was reached after realising that there were no new themes emerging and no new information generated in the already identified themes from further responses obtained.

### Implications for practice, policy and research

Deaths of children under five arising from knowledge deficit are avoidable through health education, thought to be easier to accomplish through emerging technologies. This study’s findings could be used as a basis for introducing ETs in Makonde’s public health facilities to enhance paediatric care. Policymakers and health authorities in Zimbabwe must help address the identified challenges by introducing emerging technologies that can help public health workers in Makonde District to conduct health education, aid diagnosis, deliver critical supplies on time and minimise the need for referrals to the provincial hospital. The district only has one government hospital, Chinhoyi Provincial Hospital, which acts as the referral one. Policy could look into providing facilitating conditions for ETs, such as training and infrastructure. Future studies could focus on evaluating ETs’ effectiveness and investigating what could affect the ETs introduction to promote under-five healthcare in Makonde District, given that the health workers are receptive to the technologies.

## Conclusion

SSA has high U5MR due to challenges like limited access to care, knowledge deficit, understaffing and resource shortages. Without addressing these challenges, SSA will likely miss the UN SGD 3 target. The study explored public health workers’ perceptions of emerging technologies’ role in reducing U5MR in a low-resource setting in Zimbabwe. The perceived role includes health education, minimal loss to follow-up, enhanced QoC, remote diagnosis, reduced misdiagnosis, drug supply management and skills development. Findings provide policy directions for technology-driven paediatric care and digital interventions to end preventable neonatal and under-five mortalities.
